# Prosthodontic Rehabilitation of a Bruxer Patient with Severely Worn Dentition: A Clinical Case Report

**DOI:** 10.5681/joddd.2009.008

**Published:** 2009-03-16

**Authors:** Farhang Mahboub, Elnaz Moslehi Fard, Farideh Geramipanah, Habib Hajimiragha

**Affiliations:** ^1^Assistant Professor, Department of Prosthodontics, Faculty of Dentistry, Tabriz University of Medical Sciences, Iran; ^2^Associate Professor, Department of Prosthodontics, Faculty of Dentistry, Tehran University of Medical Sciences, Iran; ^3^Assistant Professor, Department of Prosthodontics, Faculty of Dentistry, Tehran University of Medical Sciences, Iran

**Keywords:** Metal-ceramic restoration, occlusal plane, occlusal vertical dimension (OVD), prosthodontic rehabilita-tion, tooth wear

## Abstract

The management of tooth wear has been a subject of increasing interest from both preventive and restorative points of view. This article describes the full mouth rehabilitation of a 54-year-old bruxer woman with a severely worn dentition and other dental problems including unsuitable restorations and several missing teeth. The treatment entailed using cast posts and cores, metal-ceramic restorations, and a removable partial denture. As with the treatment procedure of such cases, equal-intensity centric occlusal contacts on all teeth and an anterior guidance in harmony with functional jaw movements were especially taken into account.

## Introduction

Severe tooth wear is a potential threat for dentition and masticatory function. Many factors may combine to produce the worn dentition and the etiology often remains unidentified.^1^ Tooth wear has been classified into the following four types: (1) attrition, that is the wear of teeth or restorations caused by tooth to tooth contact during mastication or parafunction; (2) abrasion, that is the loss of tooth surface caused by abrasion with foreign substances other than tooth to tooth contact; (3) erosion, that is the loss of tooth surface by chemical processes not involving bacterial action; and (4) abfraction, that is non-carious cervical wedge-shaped defect caused by occlusal stresses.^2-4^ The management of tooth wear, especially attrition, is becoming a subject of increasing interest in the prosthodontics literature, from both preventive and restorative points of view.^5^ A critical aspect for successful treatment is to determine the occlusal vertical dimension (OVD) and the inter-occlusal rest space (IRS). A systematic approach for managing tooth wear can lead to a predictable and favorable prognosis.^6^

This article presents the stages of prosthodontic rehabilitation, from diagnosis to final treatment and follow-up, of a bruxer patient with severely worn dentition, some extracted teeth and uneven occlusal plane, using cast posts and cores, metal-ceramic restorations, mandibular removable partial denture (RPD) and an occlusal splint for protecting the restorations from patient’s parafunction.

## Case Report

### Examination

A 54-year-old woman was referred to the Department of Prosthodontics in the Faculty of Dentistry at Tehran University of Medical Sciences, Iran, for prosthodontic treatment. The patient’s chief complaint was the restoration of the worn teeth, in addition to the replacement of unacceptable restorations and missing teeth. An initial evaluation of the patient indicated a history of depression, and also, parafunctional habits of bruxism and clenching. Oral hygiene was fair and there was no periodontal problem. Clinical and radiographic examinations and diagnostic casts revealed severe attrition and abfraction, especially on anterior teeth and an uneven occlusal plane (Figure 1a,b). The causes of the severe wear were parafunctional habits, unsuitable restorations, and a lack of left posterior occlusion due to missing left mandibular posterior teeth.


Figure 1. Frontal view of teeth in occlusion (a), and panoramic radiograph (b) before treatment.

**a:**
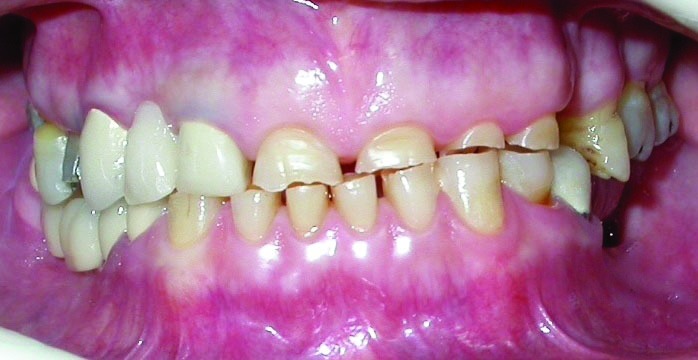

**b:**
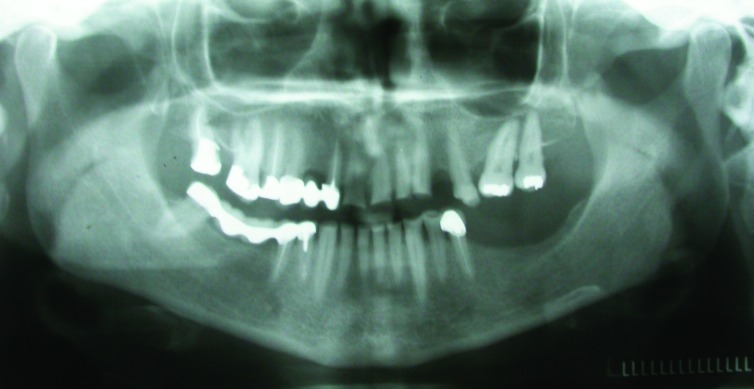

### Treatment

**Figure 3:**
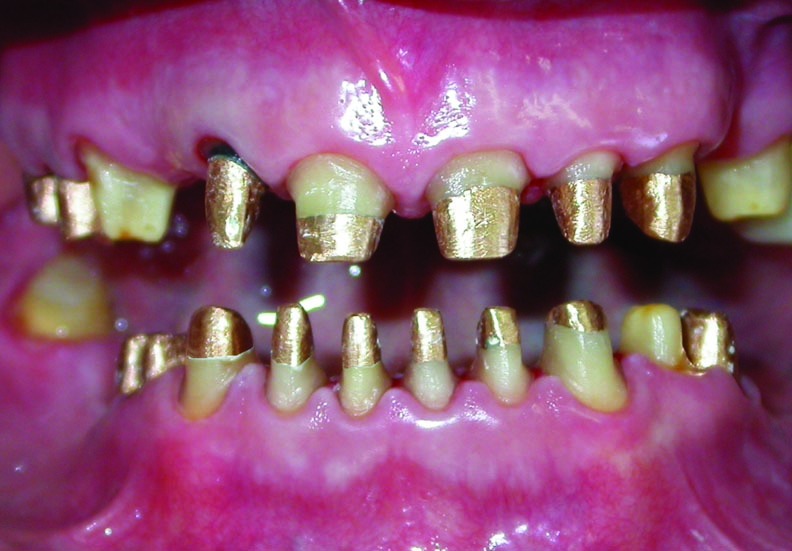
Cast gold posts and cores.


After oral hygiene instructions and removing the existing restorations, making impressions and diagnostic work-up, removable provisional prostheses were fabricated with correct occlusal plane and adjusted clinically for achieving good esthetics, phonetics and OVD. This removable prosthesis was used to evaluate the OVD and patient tolerance (Figure 2). Root canal therapy (RCT) of worn teeth and re-treatment of teeth with unacceptable RCTs were performed and cast gold posts and cores were fabricated (Figure 3). Removable provisional prostheses were replaced with fixed provisional restorations after two months. These restorations were fabricated according to the diagnostic wax-up (Figure 4), for which the Broadrick flag analyzer was used to determine the curve of occlusal plane. Impressions were made from provisional restorations, and casts were transferred to the Denar Mark II articulator (Teledyne Water pik, Fort Collins, CO, USA) using the Denar Slidematic facebow (Teledyne Water pik, Fort Collins, CO, USA) and mandibular record base. Then, an anterior guide table was customized by pattern resin (Duralay, Reliance Dental MFG Co, Worth, CO, USA).


Figure 2. Maxillary (a) and mandibular (b) removable provisional prostheses in place.

**a:**
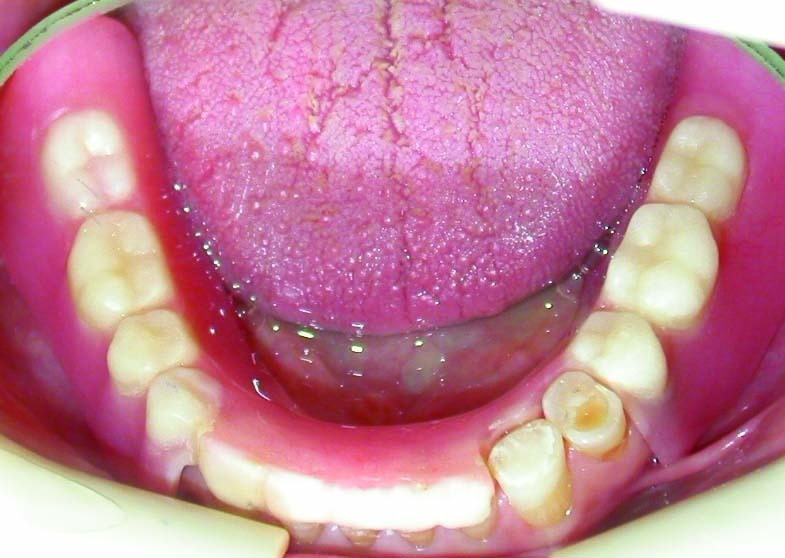

**b:**
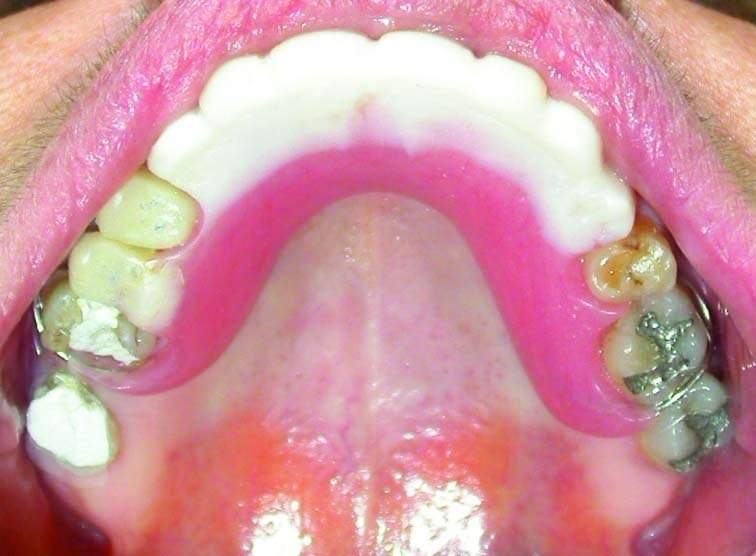

Figure 4. Diagnostic wax up (a) and fixed provisional restorations in place (b).

**a:**
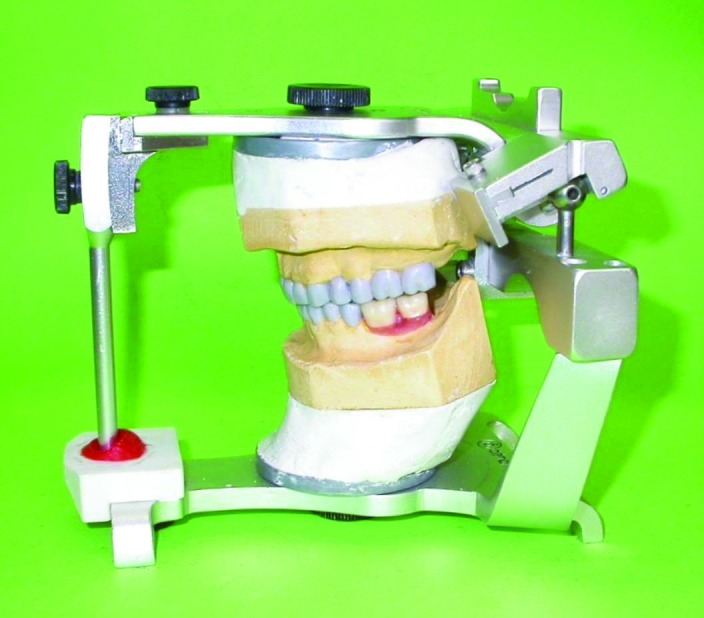

**b:**
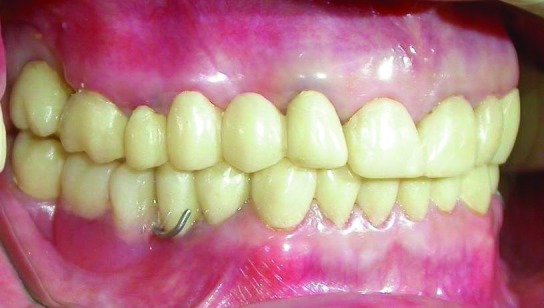

After completion of teeth preparations, the final impressions were made with silicon impression material (Speedex, Coltene AG, Alstatten, Switzerland) and metal-ceramic restorations were fabricated. In mandibular restorations, rest seats, guide planes and retentive undercuts were formed in the RPD abutments. Kennedy class II mode 1 mandibular RPD was fabricated using altered cast technique and delivered (Figure 5). Finally, the occlusion of restorations was adjusted so that equal-intensity centric contacts were established on all teeth (Figure 6; a) and anterior guidance discluded all posterior teeth in eccentric jaw movements. A maxillary occlusal splint was fabricated for protecting the restorations from patient’s parafunction. The smile view of patient after treatment is shown in Figure 6. One year follow-up showed no problem in teeth, restorations and temporomandibular joints.


Figure 5. Occlusal view of the maxillary (a) and mandibular (b) arch after treatment.

**a:**
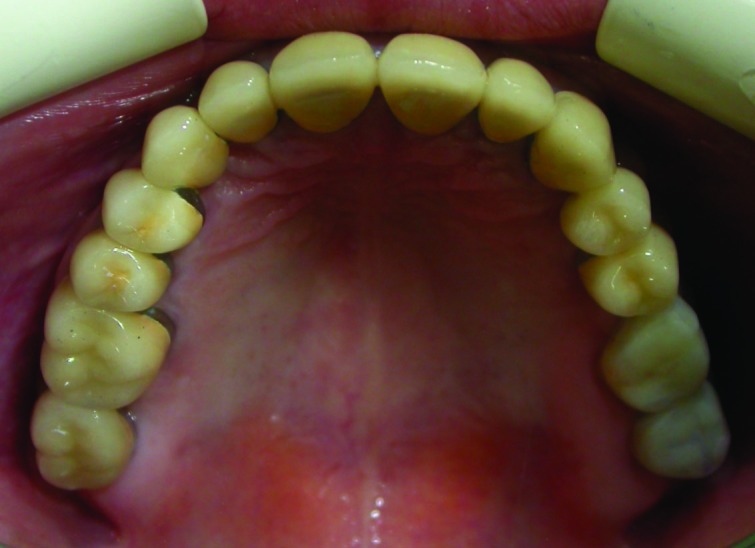

**b:**
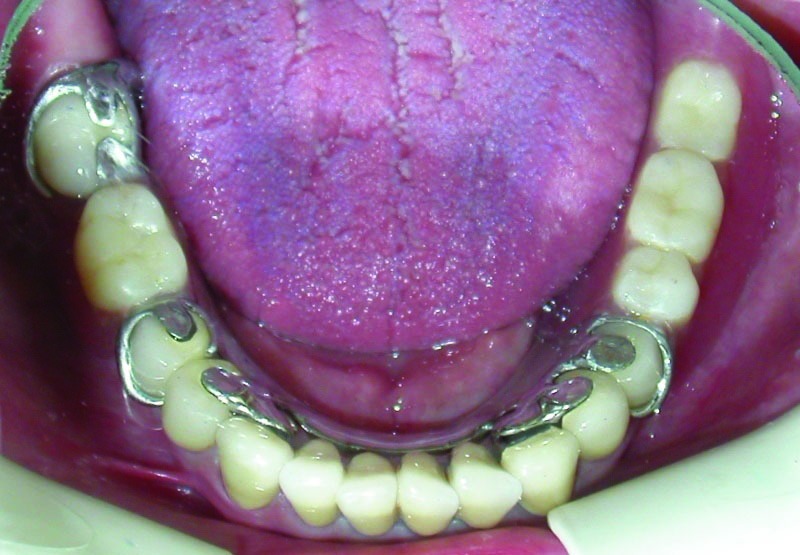


Figure 6. Frontal view of teeth in occlusion after treatment (a); smile view after treatment (b).

**a:**
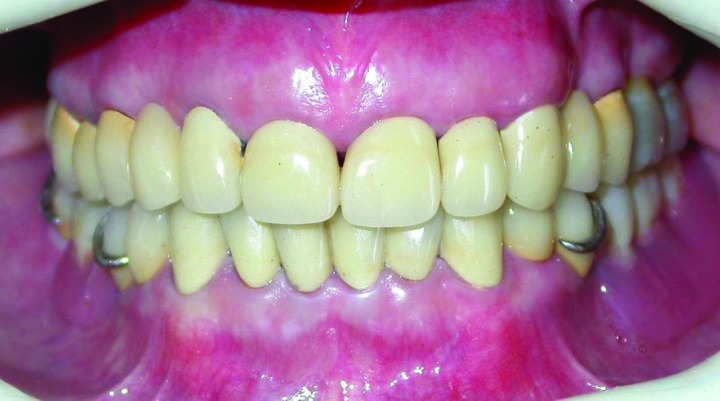

**b:**
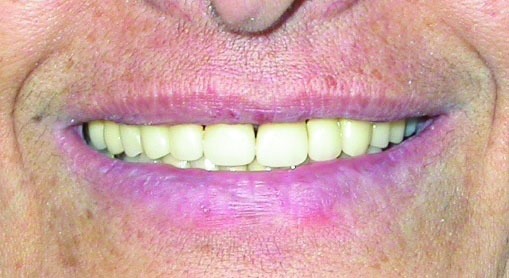

## Discussion

In the treatment of severely worn teeth, equal-intensity centric occlusal contacts on all teeth should be accomplished. An anterior guidance should also be established in harmony with normal functional jaw movements and all posterior teeth discluded immediately during any eccentric jaw movement. If there is habitual bruxism, an occlusal splint should be delivered to the patient.^7^
